# Insecticidal Activities of Chloramphenicol Derivatives Isolated from a Marine Alga-Derived Endophytic Fungus, *Acremonium vitellinum*, against the Cotton Bollworm, *Helicoverpa armigera* (Hübner) (Lepidoptera: Noctuidae)

**DOI:** 10.3390/molecules23112995

**Published:** 2018-11-16

**Authors:** Dan Chen, Peng Zhang, Tong Liu, Xiu-Fang Wang, Zhao-Xia Li, Wei Li, Feng-Long Wang

**Affiliations:** 1 College of Plant Protection, Shenyang Agricultural University, Shenyang 110866, China; chendan@caas.cn; 2 Tobacco Research Institute of Chinese Academy of Agricultural Sciences (CAAS), Qingdao 266101, China; zhangpeng@caas.cn (P.Z.); liutongdsg@126.com (T.L.); wangxiufang02@caas.cn (X.-F.W.); 3 College of Marine Life Sciences, Ocean University of China, Qingdao 266003, China; zhaoxiali0503@163.com (Z.-X.L.); liwei01@ouc.edu.cn (W.L.)

**Keywords:** alga-derived endophytic fungus, *Acremonium vitellinum*, secondary metabolites, insecticidal activities, molecular mechanism

## Abstract

A great deal of attention has been focused on the secondary metabolites produced by marine endophytic fungi, which can be better alternatives to chemicals, such as biopesticides, for control of polyphagous pests. On the basis of its novel biocontrol attributes, chemical investigation of a marine alga-derived endophytic fungus, *Acremonium vitellinum*, resulted in the isolation of three chloramphenicol derivatives (compounds **1**–**3**). Their chemical structures were elucidated by detailed analysis of their nuclear magnetic resonance spectra, high-resolution electrospray ionization mass spectrometry, and by comparison with the data available in the literature. In this paper, compound **2** was firstly reported as the natural origin of these fungal secondary metabolites. The insecticidal activities of compounds **1**–**3** against the cotton bollworm, *Helicoverpa armigera*, were evaluated. The natural compound **2** presented considerable activity against *H. armigera*, with an LC_50_ value of 0.56 ± 0.03 mg/mL (compared to matrine with an LC_50_ value of 0.24 ± 0.01 mg/mL). Transcriptome sequencing was used to evaluate the molecular mechanism of the insecticidal activities. The results presented in this study should be useful for developing compound **2** as a novel, ecofriendly and safe biopesticide.

## 1. Introduction

*Helicoverpa armigera* (Hübner) is recognized as a polyphagous and cosmopolitan insect pest and has a high damage potential for various economically important crops around the world, including cotton, corn, tomatoes, sorghum, soybeans, and tobacco [1]. Some biological characteristics, such as polyphagy, mobility, fecundity, and facultative diapauses, can increase the survival and population outbreaks of the pest in agroecosystems [2]. These pests, which attack more than 150 different host species, are considered as the most economically important insect pests in many countries, such as India, Japan, China, and Southeast Asia [3]. Owing to their biological characteristics and high damage potential, effective prevention and control of these polyphagous pests becomes a challenging work in agricultural fields.

Currently, the prevention and control of *H. armigera* is largely dependent on chemical pesticides [4]. However, total reliance on the application of synthetic insecticides to control *H. armigera* has not achieved the desired success, and has resulted in the emergence of pesticide resistance, environmental contamination, toxicity to non-target organisms, disruption of ecological balance, and health hazards [5]. Thus, many attempts have been made to find alternate methods for its control. The use of resistant compounds of natural origin has proven to be an appropriate alternative for the control of *H. armigera* [3,5,6].

Marine-derived microorganisms have shown promising potential in the production of diverse bioactive metabolites. Endophytic fungi from various marine algal hosts are increasingly being considered as sources of pharmaceutical compounds [7]. Numerous secondary metabolites with a wide range of anticancer, antibiotic, antioxidative, and insecticidal activities have been isolated and identified. Among them, the compounds with significant mortality to pests have attracted the attention of agricultural scientists [8,9]. In this study, a fungal strain, *Acremonium vitellinum*, was isolated from the fresh inner tissue of an unidentified marine red alga. Chemical investigation of this fungus led to the isolation of three chloramphenicol derivatives (compounds **1**–**3**). Herein we report the isolation, biological evaluation, and molecular mechanism of the isolated compounds.

## 2. Results and Discussion

### 2.1. Elucidation of the Structure of Isolated Compounds ***1**–**3***

**Figure 1:**
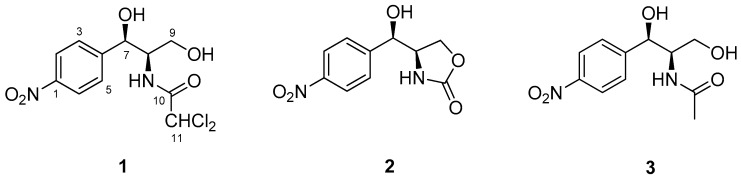
Structures of the natural compounds **1**–**3,** isolated from *Acremonium vitellinum*.

The ethyl acetate (EtOAc) extracts of *A. vitellinum* culture showed considerable activity against *H. armigera* in a preliminary screening assay (with an LC_50_ value of 1.8 ± 0.3 mg/mL), thus prompting us to perform an in-depth analysis of the bioactive secondary metabolites present in it. Thereafter, a large-scale fermentation of this fungus was performed in liquid medium, which lead to the isolation of three chloramphenicol derivatives (compounds **1**–**3**) (Figure 1).

Compound **1** was initially isolated as a white amorphous powder, and its molecular formula was assigned as C_11_H_12_Cl_2_N_2_O_5_ by high resolution electrospray ionization mass spectroscopy (HRESIMS) at *m*/*z* 323.0196 [M + H]^+^ (calcd 323.0196). Compound **1** was identified as chloramphenicol based on its spectroscopic data (Table 1) and by comparison of its physical properties with those reported in the literature. Chloramphenicol, first isolated from *Streptomyces venezuelae* in 1947 [10], is a natural antibiotic with a relatively broad spectrum of antimicrobial activity [11]. The structure of compound **2** was elucidated by comparison of its NMR data with those reported previously in the literature. Compound **2** contained an oxazolidin-2-one moiety synthesized by a catalytic method for the cyclization of carbamates [12]. Compound **3** showed an acetamide signal (δ_H/C_ 1.69/22.8) in its NMR spectrum (Table 1) as a substitute for a dichloroacetamide signal, which is observable in chloramphenicol [13]. It should be pointed out that compound **2** was previously reported as a synthetic product [12], and herein, the isolation of this fungal secondary metabolite from a natural source is reported for the first time.

### 2.2. Insecticidal Activities of the Isolated Compounds

The isolated compounds **1**–**3** and commercial matrine were tested for their larvicidal activity against the 3rd instar larvae of *H. armigera*. The natural compound **2** showed certain insecticidal activity against *H. armigera*, with an LC_50_ value of 0.56 ± 0.03 mg/mL, whereas compounds **1** and **3** exhibited weak activities, with LC_50_ values of 0.93 ± 0.05 and 0.91 ± 0.06 mg/mL, respectively. The LC_50_ value of the positive control, matrine, was 0.24 ± 0.01 mg/mL.

### 2.3. Effects of Compound ***2*** on the Activities of GST, CAT, AChE and T-AOC in H. armigera

As compound **2** showed the most promising insecticidal activity, its effects on the activities of glutathione S-transferase (GST), catalase (CAT), and acetylcholinesterase (AChE) as well as on the total antioxidant capacity (T-AOC) in *H. armigera* were evaluated. The protective and detoxifying enzymes play an important role in insects in the metabolism of foreign toxic substances [14]. GST is an important detoxification enzyme in insects, which can catalyze the conjugation of GSH to xenobiotic compounds and enables their metabolization out of the cell/body [15]. CAT, the main protective enzyme in insects, can decompose H_2_O_2_ [16]. T-AOC is a comprehensive index of the functional status of various antioxidants in insects [17,18]. Under normal conditions, free radicals in cells are in a dynamic equilibrium, and are maintained at low levels to prevent toxicity to the organisms. When the protective system of enzymes is damaged, the concentration of oxygen free radicals in biological organisms increases, resulting in threat to the biological functions of cells. The changes in the enzyme activities may reflect differences in the degree of metabolism. AChE is a key enzyme in the central nervous system of insects [19].

As shown in Table 2, the GST activity in the 0.1 mg/mL treatment showed no significant difference compared to that in the control group after 48 h of exposure (*p* < 0.05). However, the GST activity in the 0.5 mg/mL treatment was significantly lower than that in the 0.1 mg/mL treatment and the control groups. This result indicated that compound **2** had inhibitory effects on the GST activity of *H. armigera*. For CAT activity, significant decreases were observed in the 0.1 and 0.5 mg/mL treatments after 48 h of exposure (*p* < 0.05), which implied that compound **2** caused oxidative stress in *H. armigera*. Significant increases in T-AOC were observed in the 0.1 and 0.5 mg/mL treatments after 48 h of exposure (*p* < 0.05). The AChE activity changed more significantly than the activities of other enzymes. In the 0.1 mg/mL treatment, the AChE activity was 1.6 times higher than that in the control group, whereas in the 0.5 mg/mL treatment, it was 5.1 times higher than in the control group.

The activities of GST and CAT were significantly inhibited, indicating that compound **2** could inhibit the activity of these detoxifying and protective enzymes after entering into the larva of *H. armigera*, making it difficult to be metabolized, and thus, enhancing the toxicity to *H. armigera.* Some studies had shown that the activities of GST and CAT in the insect were inhibited after treatment by pesticides [20]. Besides antioxidant enzymes, insects also have other antioxidant mechanisms, such as trehalose and α-tocopherol, which also play an active role in oxidative stress [21,22]. The increase in T-AOC upon exposure of *H. armigera* to compound **2** might be because of the compensatory response of the body’s antioxidant system, as a result of stimulation by compound **2**. The increased activity of AChE showed that compound **2** had an effect on the acetylcholine receptor of *H. armigera*. AChE is a target of inhibition by insecticides [19]. There are lots of studies showing the inhibition of cholinesterase activities by pesticides, whereas some studies have shown that the exposure of insects to pesticides can increase the activity of AChE [23,24]. The increased AChE activity may be related to the induction of the defense mechanism of insects when feeding on foreign toxic substances.

### 2.4. Molecular Mechanism of Action of Compound ***2***

#### 2.4.1. Analysis of Differentially Expressed Genes

**Figure 2:**
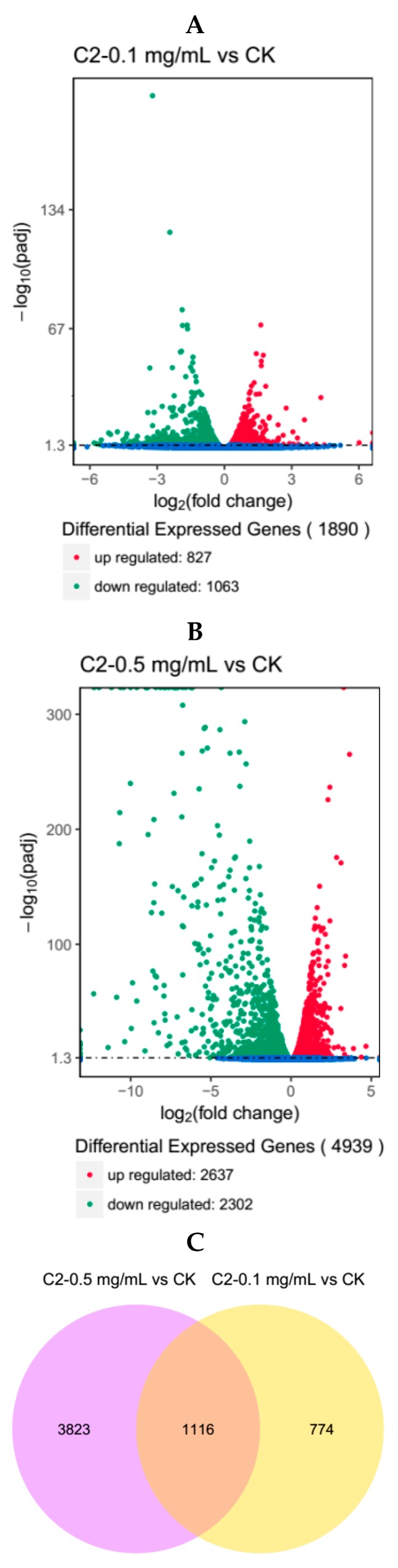
Bioinformatic analysis of differentially expressed genes (DEGs). Volcanic figures of DEGs in the (**A**) C2-0.1 mg/mL vs. CK and (**B**) C2-0.5 mg/mL vs. CK comparisons; (**C**) Venn diagram showing the number of genes expressed in the two groups.

Differential analysis of gene expression was performed on samples from the three groups. The RNA-seq data of C2-0.1 mg/mL vs. CK revealed that the number of differentially expressed genes (DEGs) in the larvae of *H. armigera* was 1890, of which 827 genes were up-regulated and 1063 were down-regulated (Figure 2A). The RNA-seq data of C2-0.5 mg/mL vs. CK revealed that the number of DEGs in the larvae of *H. armigera* was 4939, of which 2637 genes were up-regulated and 2302 were down-regulated (Figure 2B). Furthermore, the results indicated that compound **2** had an effect on *H. armigera*, which increased with its concentration.

The data for the DEGs identified in the C2-0.1 mg/mL vs. CK and C2-0.5 mg/mL vs. CK comparisons were analyzed by Venn diagram. Of the 1890 differentially expressed genes in C2-0.1 mg/mL, 1116 were also differentially expressed in C2-0.5 mg/mL compared to their expression in CK (Figure 2C).

#### 2.4.2. GO Enrichment Analysis of DEGs

To further illustrate the functional enrichment of DEGs between the different treatment samples, we used the GOseq method [25], which is based on Wallenius non-central hyper-geometric distribution, to perform a significant enrichment analysis of the gene ontology (GO) terms. The results showed that these DEGs were closely related to biological processes, molecular functions, and cellular components. A total of 30 GO terms with the most significant enrichment were selected from each group.

**Figure 3:**
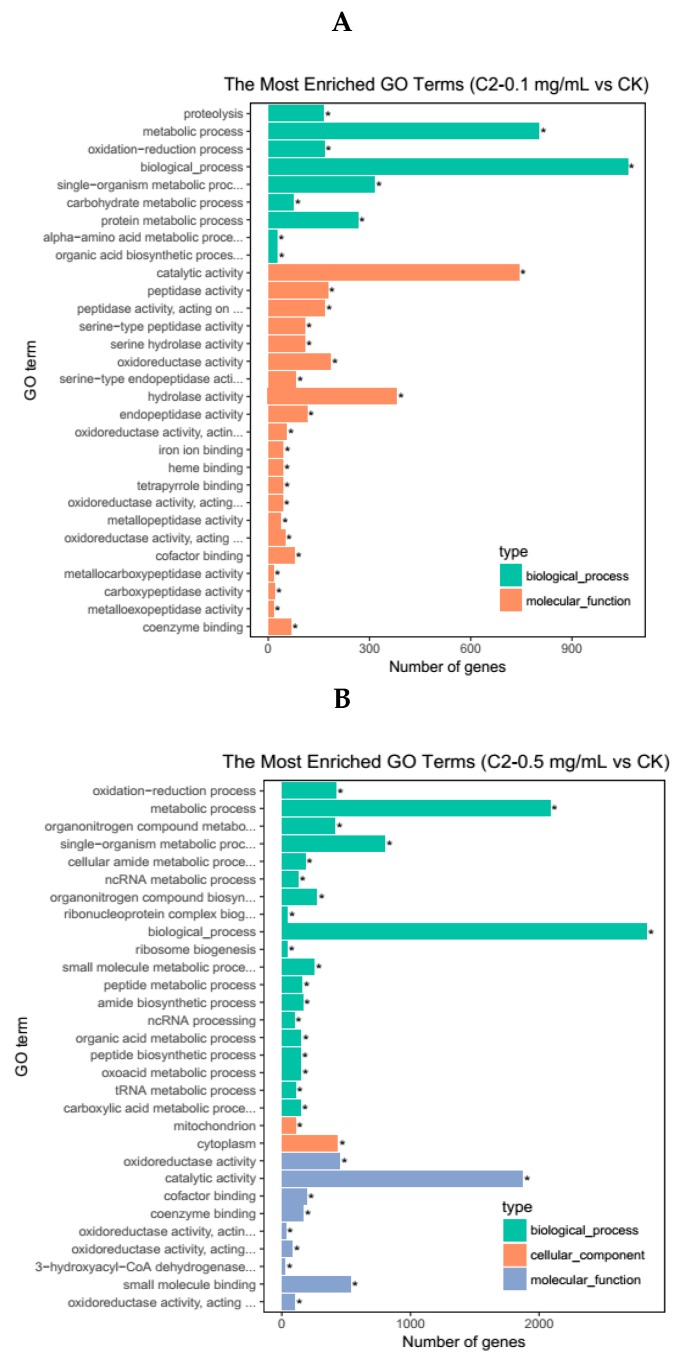
Functional annotation of differentially expressed genes using the gene ontology (GO) database. (**A**) C2-0.1 mg/mL vs. CK; (**B**) C2-0.5 mg/mL vs. CK.

The DEGs obtained in the C2-0.1 mg/mL vs. CK comparison were subjected to GO annotation analysis (Figure 3A). In the figure, nine GO terms are shown to belong to the biological process category. Among them, more than 300 DEGs were in the metabolic process terms, biological process terms, and single-organism metabolic process terms. This result indicated that compound **2** had a more severe effect on the metabolic process of *H. armigera* than the control compound (acetone). Twenty-one GO terms belonged to the molecular function category, in which the number of DEGs in the catalytic activity term and hydrolase activity term, exceeded 300.

The DEGs obtained in the C2-0.5 mg/mL vs. CK comparison were subjected to GO annotation analysis (Figure 3B). Among these DEGs, 19 belonged to the biological process category, while in the metabolic process and biological process categories, the number of DEGs exceeded 1000. Two GO terms belonged to the cell component category and nine GO terms belonged to the molecular function category, in which the number of DEGs in the catalytic activity term was 1871. The results of the two treatment groups showed that compound **2** significantly affected the metabolic process, biological process, and the catalytic activity terms. When foreign toxic substances enter the insect body, they undergo various metabolic reactions, such as oxidation, hydrolysis, and conjugation, catalyzed by various enzymes and produce corresponding metabolites [26]. When metabolic processes and catalytic activities are affected, toxic substances accumulate in the insect body, which kill the insects.

#### 2.4.3. KEGG Analysis of DEGs

To investigate the biological pathways associated with significantly different genes between the different treatments, we used the Kyoto Encyclopedia of Genes and Genomes (KEGG) database to map pathways in which DEGs from the three sample groups (C2-0.1 mg/mL vs. CK, C2-0.5 mg/mL vs. CK) might be, or were involved. For analysis, each group was compared to the scatter plot of the top 20 from the KEGG enrichment analysis (the top 20 of the most significantly enriched pathways).

**Figure 4:**
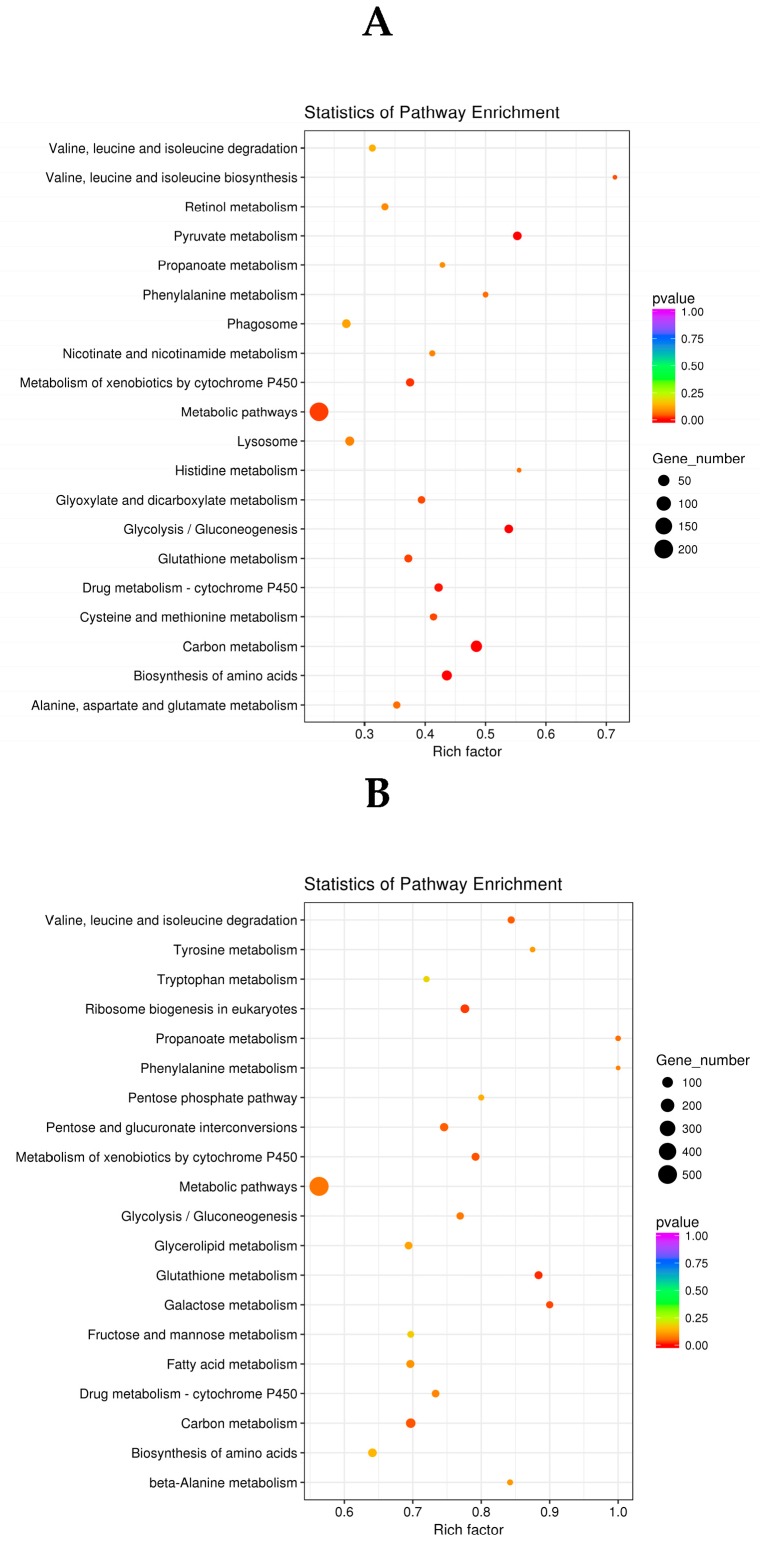
Functional annotation of differentially expressed genes using the KEGG database. (**A**) C2-0.1 mg/mL vs. CK; (**B**) C2-0.5 mg/mL vs. CK.

The KEGG pathway enrichment analysis of the differential genes in the C2-0.1 mg/mL vs. CK samples revealed that metabolic pathways contained the largest number of differential genes. Other pathways for pesticide metabolism and insecticide targets included drug metabolism-cytochrome P450, glutathione metabolism, and metabolism of xenobiotics by cytochrome P450 [27,28] (Figure 4A). Among them, cytochrome P450 is a key enzyme that metabolizes toxic substances [29] and plays an important role in the metabolism of secondary compounds and insecticides in insects. As an important binding element of glutathione synthesis [30], glutamate and its downstream product, the inhibitory neurotransmitter gamma-aminobutyric acid (GABA), are closely related to insect detoxification [31]. The effects of metabolic pathways, such as P450 and glutathione metabolism, indicate that the detoxification ability of *H. armigera* was inhibited.

The KEGG pathway enrichment analysis of the DEGs in the C2-0.5 mg/mL vs. CK comparison revealed that the number of DEGs related to metabolic pathways drug kibone-cytochrome P450, glutathione metabolism, metabolism of xenobiotics by cytochrome P450, and rich factor, increased with the increase in concentration compared to that in the C2-0.1 mg/mL vs. CK comparison (Figure 4B). The increase in the number of DEGs and in the rich factor indicated that at higher concentration, the compounds further affected multiple detoxification metabolic pathways, and the detoxification capacity of *H. armigera* was more seriously damaged.

#### 2.4.4. Differentially Expressed Genes Associated with the Insecticide Target

We compared the differential genes obtained from the three sample groups (C2-0.1 mg/mL vs. CK, C2-0.5 mg/mL vs. CK) and screened for differential genes associated with common insecticide targets [32] (Table 3). The original data are shown in the Supplementary Materials (Tables S1 and S2). It can be seen from the differential expression of common genes targeted by insecticides [32] that compound **2** mainly affected the NADH dehydrogenase of *H. armigera*, and the number of DEGs of NADH dehydrogenase was two at low concentration, whereas it was 19 at high concentration. The function of NADH dehydrogenase is to transfer electrons from NADH to ubiquinone. Moreover, it is responsible for transferring four protons from the mitochondrial membrane to the outer membrane. When NADH dehydrogenase is inhibited, the electron transport chain of insect cells is inhibited and eventually causes the death of insect [33]. In addition to the influence on NADH dehydrogenase, compound **2** also affected acetylcholinesterase and acetylcholine. With the increase in the concentration of compound **2**, the gamma-aminobutyric acid receptor was also affected.

#### 2.4.5. Differentially Expressed Genes Associated with Digestive, Detoxification, and Protective Enzymes

In the midgut of lepidopteran insects, serine proteases, such as trypsin and chymotrypsin, are mainly used for digestion. Compared to CK, the number of DEGs of trypsin and chymotrypsin increased in the samples treated with different concentrations of compound **2** (Table 4). The original data were shown in the Supplementary Materials (Tables S1 and S2). With the increase in concentration, lysosomed were also affected. It is indicated that compound **2** had an effect on the digestive system of *H. armigera*. Moreover, with the increase in concentration, catalase, which is a protective enzyme in *H. armigera*, may also be destroyed. As a result, the concentration of oxygen free radicals in *H. armigera* might have been too high, and the cell function may have been threatened.

The differential genes associated with detoxification enzymes, carboxylesterase, glutathione S-transferase, cytochrome P450 and the DEGs related to detoxification increased with the increase in concentration of compound **2**, indicating that the detoxification ability of metabolic enzymes was greatly affected. Moreover, the number of DEGs of cytochrome P450 in the different control groups was much higher than that of carboxylesterase and glutathione S-transferase. This is inextricably linked to the extremely important role of cytochrome P450 enzymes in the metabolism of insects.

In addition to the direct killing effect of insecticidal substances, the biological, ecological behavior, fertility, detoxification, and metabolism of the surviving individuals are affected to different degrees by exposure to different amounts of the compound, and with the passage of time [34]. The toxicological effects of compound **2** were determined by measuring the changes in the protective and detoxification enzymes present in *H. armigera*, and transcriptome sequencing was performed to analyze the differential expression of the genes. The elucidation of the mechanism of the insecticidal effect of compound **2** on *H. armigera* as described in the present study hold a huge significance for controlling this pest. On the other hand, the Agrobacterium-mediated transformation with an insecticidal protein gene needs to be further studied. Chloramphenicol derivatives are known for certain cytotoxicity, thus the cytotoxicity and other factors such as the environmental impact, as well as the potential side effects of these compounds also need to be further investigated.

## 3. Materials and Methods

### 3.1. General Experimental Procedures

Optical rotations were measured on a P-1020 digital polarimeter (Jasco, Tokyo, Japan). The ^1^H-, ^13^C-, and 2D-NMR spectra were acquired using an Agilent DD2 500 MHz NMR spectrometer (500 MHz for ^1^H and 125 MHz for ^13^C), with TMS as an internal standard (Agilent, Santa Clara, CA, USA). Analytical HPLC was performed on a Waters UPLC-class system (Waters, Milford, MA, USA), equipped with a TUV-detector, using a C_18_ column (1.6 μm, 2.1 mm × 50 mm). Column chromatography (CC) was performed using silica gel (100–200, 200–300 mesh, Qingdao Haiyang Chemical Factory, Qingdao, China), Lobar LiChroprep RP-18 (40–60 μm, Merck, Darmstadt, Germany), and Sephadex LH-20 (Merck) columns. All the solvents used were of analytical grade.

### 3.2. Fungal Material

The fungus, *Acremonium vitellinum*, was isolated from the fresh inner tissue of an unidentified marine red alga, which was collected from Qingdao, China, in June 2016. After surface sterilization, the isolated strain was considered as an endophyte [7]. This strain was preserved at Tobacco Research Institute, Chinese Academy of Agricultural Sciences, and the GenBank (NCBI) accession number (MH726097) was obtained.

### 3.3. Fermentation, Extraction and Isolation

For chemical investigations, the fungus was statically cultivated in a 1 L Erlenmeyer flask containing 300 mL of the potato dextrose broth (PDB) with 2% mannitol, 1% glucose, 0.3% peptone, 0.5% yeast extract, and 300 mL seawater (100 flasks) for 30 days at 28 °C. The fermented broth (60 L) was extracted repeatedly with EtOAc, whereas the mycelia (128.3 g) were extracted three times with a mixture of acetone and H_2_O (80–20%). They were then combined to obtain the residue (45.6 g), which was subjected to silica gel chromatography using a VLC column, with a stepwise gradient of a mixture of petroleum ether (PE):ethyl acetate (EtOAc) (from 5:1 to 1:1), to collect five fractions (Fr.1−Fr.5). The bioactivity-guided isolation indicated that Fraction 4 (PE:EtOAc = 2:1) had the strongest insecticidal activity. Thus, we chose this fraction for further isolation. Fraction 4 (7.2 g), eluted with PE:EtOAc (2:1, *v*/*v*), was purified by CC (silica gel, CHCl_3_–MeOH gradient, from 50:1 to 10:1) to obtain five subfractions (Fr. 3.1−Fr. 3.5). Fr. 3.3 (1.2 g) was further separated by Lobar LiChroprep RP-18 from MeOH:H_2_O (3:7 to 7:3), and finally using Sephadex LH-20 (MeOH) to obtain compounds **1** (62.0 mg), **2** (160 mg), and **3** (56.8 mg).

### 3.4. Insecticidal Assay

The artificial insect mixed drug method [35] was used to determine the insecticidal activity of the isolated compounds. Compounds **1**–**3** were formulated into mother liquors with acetone, and the mother liquors were sequentially added to the artificial diets to obtain 0.1, 0.2, 0.4, 0.6, 0.8, and 1.0 mg/mL of toxic feed, respectively. The feed was placed in a 24-well plate, and the 3rd instar larvae of *H. armigera* showing uniform growth were selected; one test insect was inserted into each well. Each treatment was repeated three times with 20 replicates per turn. Commercial matrine (Sigma-Aldrich, St. Louis, MO, USA) served as a positive control, acetone treatment was used as a blank control (CK) [36], and the final concentration of acetone in each treatment did not exceed 1% (by volume) [37]. The treated test insects were kept and observed under the set conditions of temperature (25 ± 1 °C), relative humidity (RH; 70% ± 5%), and photoperiod (L:D = 16:8 h). The number of larval deaths was counted after 48 h; the test insects were touched with a fine brush, and no reaction or only slight reaction (but could not crawl normally) was considered as an indication of death. The data were statistically analyzed using SPSS software (Version 18.0, SPSS Inc.) to determine the LC_50_ values.

### 3.5. Enzyme Assays

#### 3.5.1. Enzyme Preparation

Based on the results of virulence, the feed was mixed with compound **2** at 0.1 and 0.5 mg/mL and administered for 48 h; acetone treatment was used as a blank control (CK), the feeding conditions were the same as described in Section 3.4. Approximately 0.1 g of the selected 3rd instar larvae of *H. armigera* was homogenized in 1.0 mL extraction buffer (0.1 M phosphate buffer with 1% Triton-X-100 pH 7.8 for AChE; 0.1 M phosphate buffer pH 7.0 for GST; 0.1 M phosphate buffer pH 7.8 for CAT, T-AOC and the protein) on ice bath and centrifuged at 8000× *g* for 10 min at 4 °C. The supernatant was collected and kept on ice for further use.

#### 3.5.2. Activity Assays

The activities of GST, CAT, T-AOC, and AChE and the protein concentrations were determined using commercially available assay kits (Suzhou Comin Biotechnology Co., Ltd., Suzhou, China) [38] and a fluorescence spectrophotometer (F-4600, Hitachi High-Technologies Corp., Tokyo, Japan).

GST catalyzes the conjugation of GSH and 1-chloro-2,4-dinitrobenzene (CDNB). The activity of GST was calculated by measuring the change in absorbance upon formation of the GSH-CDNB conjugate at 340 nm. One unit of GST activity was defined as the amount that catalyzed the conjugation of 1 μmol/L CDNB with GSH per minute, per milligram protein.

CAT decomposes H_2_O_2_, decreasing the absorbance of the reaction mixture at 240 nm with time. The activity of CAT was thus calculated according to the change in the rate of absorbance. One unit of CAT activity was defined as the amount that catalyzed the decomposition of 1 μmol/L H_2_O_2_ per minute, per milligram protein.

AChE catalyzes the hydrolysis of Ach to produce choline, and choline reacts with 5,5′-nitrobenzoic acid (DTNB) to produce 5-mercapto-nitrobenzoic acid (TNB). The activity of AChE was calculated by measuring the change in the rate of absorbance at 412 nm. One unit of AChE activity was defined as the amount that catalyzed 1 nmol TNB per minute per milligram protein.

Under acidic conditions, the ability of materials to reduce Fe^3+^-tripyridine triazine (Fe^3+^-TPTZ) to produce blue Fe^2+^-TPTZ reflects their T-AOC. One unit of T-AOC was defined as the standard ion concentration required to achieve the same variation in the absorbance.

The protein concentration was determined by the Coomassie brilliant blue staining method, using bovine serum albumin as the standard [39].

#### 3.5.3. Statistical Analysis

The data were statistically analyzed using SPSS software (Version 18.0, Chicago, IL, USA). The data were expressed as the means ± SD. One-way analysis of variance along with the LSD test was performed for multiple comparisons at the *p* < 0.05 level.

### 3.6. Transcriptomic Analysis

#### 3.6.1. Insect Treatments

Based on the results of virulence, the feed was mixed with compound **2** at 0.1 and 0.5 mg/mL and was administered for 48 h; acetone treatment was used as a control, the feeding conditions were the same as described in Section 3.4. The 3rd instar larvae of the *H. armigera* were selected and quickly frozen in liquid nitrogen for extraction of total RNA and RNAseq analysis [40].

#### 3.6.2. Library Construction

Total RNA from each treated sample of *H. armigera* was extracted using TRIzol reagent (Ambion, Foster City, CA, USA). The quality of the RNA samples was measured by electrophoresis on a 1% agarose gel. The purity of RNA was determined using a NanoDrop 2000 (Thermo Fisher Scientific, San José, CA, USA) and the mRNA of the required quality was enriched. Subsequently, fragmentation buffer was used to break the mRNAs into short fragments. The RNA was reverse transcribed to cDNA, and the library was constructed after cDNA purification. It was then sequenced on the Illumina HiSeq 4000 platform (Illumina, Inc., San Diego, CA, USA).

#### 3.6.3. Read Mapping to the Reference Genome

Each sample produced 52.88–77.04 million (M) raw reads. After sequencing, the raw reads were filtered to obtain clean reads; the average clean reads for the three sample sets were 60213778 (CK, Acetone), 61388118 (compound **2**, 0.1 mg/mL), and 71639269 (compound **2**, 0.5 mg/mL). The filtered sequences were compared with the reference genome of *H. armigera* (//ftp.ncbi.nlm.nih.gov/genomes/Helicoverpa_armigera/) using HISAT software (2.0.4, Baltimore, MD, USA). All the short reads in the three sample sets were uniquely mapped to the reference genome of *H. armigera* and showed 73.24% to 73.75% similarity.

#### 3.6.4. Bioinformatic Analysis of RNA-seq Data

HTSeq software (v0.6.1, Heidelberg, Germany) was used to analyze the gene expression levels in each sample, using expected number of Fragments Per Kilobase of transcript sequence per Millions base pairs sequenced (FPKM). The differentially expressed genes were identified using the DESeq package (ver.2.1.0). Significantly different genes were expressed by threshold (*p* < 0.05) and |log 2 (FoldChange)| > 1.

#### 3.6.5. GO and KEGG Pathway Enrichment Analysis

The GO and KEGG pathway database were used to annotate the functions of DEGs [41,42].

#### 3.6.6. Availability of Supporting Data

The supporting data have been deposited into sequence read archive (SRA) of the National Center for Biotechnology Information (NCBI) under BioProject accession PRJNA490508 (http://www.ncbi.nlm.nih.gov/sra).

## 4. Conclusions

In this study, we isolated three chloramphenicol derivatives (**1**–**3**) from a marine alga-derived endophytic fungus, *A. vitellinum*, among which, compound **2** was found to be a new natural product. The insecticidal activities of compounds **1**–**3** were evaluated, and compound **2** exhibited the strongest activity. Using enzymatic assays, compound **2** was observed to affect the activity of the detoxification and protective enzymes of *H. armigera*, such as GST, CAT, and the affect of compound **2** on the T-AOC of *H. armigera,* resulting in an imbalance of normal physiological functions in the insects. Some studies have shown that neonicotinoid compounds can cause an increase in AChE activity [24]. In this study, compound **2** also increased the AchE activity in *H. armigera.* Thus, it was speculated that AChE might be a target of compound **2**.

We identified a number of genes related to insecticide targets and detoxification metabolism from the sequence information of *H. armigera* by screening the gene sequences. By functional prediction and annotation of these DEGs using the GO and KEGG databases, compound **2** was found to significantly affect the metabolic process, biological process, and catalytic activity terms. Compound **2** primarily effected multiple metabolic pathways associated with insecticide metabolism and insecticide target pathways. In addition, it was observed that compound **2** had an influence on the NADH dehydrogenase in *H. armigera*, inhibiting the cellular respiration, and secondarily, affecting the acetylcholine receptors and gamma-aminobutyric acid. Overall, we speculate that the mechanism of action of compound **2** in *H. armigera* might be through its effect on NADH dehydrogenase and acetylcholine receptors, through interference of the activities of related enzymes in the digestive system, and those involved in P450 and glutathione metabolism during detoxification.

## Figures and Tables

**Table 1:** ^1^H- (500 MHz) and ^13^C-NMR (125 MHz) data of compounds **1**–**3** (DMSO-*d*_6_).

	Compound 1		Compound 2		Compound 3	
No.	*δ*_H_ (mult, *J* in Hz)	*δ*_C_, type	*δ*_H_ (mult, *J* in Hz)	*δ*_C_, type	*δ*_H_ (mult, *J* in Hz)	*δ*_C_, type
1		151.7, C		149.4, C		152.5, C
2	7.58, d (8.6)	127.8, CH	7.66, d (8.6)	128.4, CH	7.58, d (8.3)	127.8, CH
3	8.14, d (8.6)	123.3, CH	8.21, d (8.6)	123.6, CH	8.16, d (8.3)	123.2, CH
4		146.9, C		147.3, C		146.7, C
5	8.14, d (8.6)	127.8, CH	8.21, d (8.6)	128.4, CH	8.16, d (8.3)	127.8, CH
6	7.58, d (8.6)	123.3, CH	7.66, d (8.6)	123.6, CH	7.58, d (8.3)	123.2, CH
7	5.05, br s	69.4, CH	4.72, t (4.0)	72.7, CH	5.00, br s	69.8, CH
8	3.92, dt (8.3, 4.2)	57.3, CH	4.02, m	57.3, CH	3.95, m	56.4, CH
9	3.58, dt (10.3, 7.2) 3.36, m	60.7, CH_2_	4.20, t (8.8) 4.11, dd (8.8, 4.6)	65.6, CH_2_	3.53, dd (14.5, 10.0) 3.28, dt (10.0, 5.1)	60.9, CH_2_
10		163.8, C		159.1, C		169.5, C
11	6.46, s	66.9, CH	-	-	1.69, s	22.8, CH_3_
7-OH	6.03, d (4.5)	-	6.06, d (4.0)	-	5.81, d (4.6)	-
8-NH	8.32, d (9.1)	-	7.74, s	-	7.56, br s	-
9-OH	4.98, t (5.4)	-	-	-	4.83, t (5.1)	-

**Table 2:** Activities of glutathione S-transferase (GST), catalase (CAT), and acetylcholinesterase (AChE) and total antioxidant capacity (T-AOC) in *H. armigera.*

	GST (μmol/min/mg prot)	CAT (μmol/min/mg prot)	T-AOC (U/mg prot)	AChE (nmol/min/mg prot)
blank control (CK)	0.332 ± 0.022 ^a^	0.111 ± 0.003 ^a^	24.394 ± 0.827 ^c^	0.555 ± 0.018 ^c^
0.1 mg/mL	0.312 ± 0.001 ^a^	0.065 ± 0.005 ^c^	38.813 ± 0.467 ^a^	0.887 ± 0.025 ^b^
0.5 mg/mL	0.267 ± 0.013 ^b^	0.080 ± 0.003 ^b^	35.517 ± 0.744 ^b^	2.828 ± 0.081 ^a^

Bars are the means ± SD of three replicates. Different letters indicate significant differences (*p* < 0.05) among the treatments.

**Table 3:** Number of pesticide targets of the differentially expressed genes.

	C2-0.1 mg/mL vs. CK	C2-0.5 mg/mL vs. CK
NADH dehydrogenase	2	19
Acetylcholinesterase	4	4
Acetylcholine	2	2
Gamma-aminobutyric acid	-	1

**Table 4:** Number of differentially expressed genes of detoxification and protective enzymes in *H. armigera.*

	C2-0.1 mg/mL vs. CK	C2-0.5 mg/mL vs. CK
Trypsin	20	20
Chymotrypsin	4	4
Lysosome	0	2
Catalase	0	2
Carboxylesterase	2	4
Glutathione S-transferase	8	22
Cytochrome P450	37	47

## Supplementary Materials

The following are available online: Table S1: Differentially Expressed Genes between C2-0.1 mg/mL vs. CK (xls), Table S2: Differentially Expressed Genes between C2-0.5 mg/mL vs. CK (xls).
