# Supplementary material for: Insecticidal Activities of Chloramphenicol Derivatives Isolated from a Marine Alga-Derived Endophytic Fungus, Acremonium vitellinum, against the Cotton Bollworm, Helicoverpa armigera (Hübner) (Lepidoptera: Noctuidae)
Source: Molecules. 2018 Nov 16;23(11):2995. doi: 10.3390/molecules23112995 (PMC6278348; doi:10.3390/molecules23112995)
Supplement: Supplementary file 1 [file molecules-23-02995-s001.pdf]

Supplementary Material

Insecticidal Activities of Chloramphenicol Derivatives Isolated from a Marine Alga-Derived Endophytic Fungus, *Acremonium vitellinum*, against the Cotton Bollworm, *Helicoverpa armigera* (Hübner) (Lepidoptera: Noctuidae)

Dan Chen [1,2], Peng Zhang[2], Tong Liu[2], Xiu-Fang Wang[2], Zhao-Xia Li[3], Wei Li[3], and Feng-Long Wang [1,2,*]

[1] College of Plant Protection, Shenyang Agricultural University, Shenyang 110161, China; chendan@caas.cn (D. C.)

[2] Tobacco Research Institute of Chinese Academy of Agricultural Sciences (CAAS), Qingdao 266101, China; zhangpeng@caas.cn (P. Z.); liutongdsg@126.com (T. L.); wangxiufang02@caas.cn (X.-F. W.)

[3] College of Marine Life Sciences, Ocean University of China, Qingdao 266003, China; zhaoxiali0503@163.com (Z.-X. L.); liwei01@ouc.edu.cn (W. L.)

***** Correspondence: wangfenglong@caas.cn (F.-L. W.); Tel: +86-532-88702117

Table of Contents

Table S1: Differentially Expressed Genes between C2-0.1 mg/mL *vs.* CK (xls)

Table S2: Differentially Expressed Genes between C2-0.5 mg/mL *vs.* CK (xls)

Table S1

Gene_id	readcount_C2-0.1 mg/mL	readcount_CK	log2FoldChange	pval	padj	Gene name	Description
1.1E+08	22680.59	29507.23	-0.37961	1.26E-05	0.0002742	LOC110370005	NADPH--cytochrome P450 reductase%2C
1.1E+08	867.7338	1430.791	-0.72149	5.59E-05	0.001019	LOC110370104	cytochrome P450 6B6-like
1.1E+08	9.447349	80.48137	-3.0907	0.0008352	0.0098845	LOC110371283	NADH dehydrogenase [ubiquinone] iron-sulfur protein 5-like
1.1E+08	17852.51	22519.54	-0.33505	0.0034974	0.031263	LOC110371343	glutathione S-transferase 1-like%2C
1.1E+08	87.05731	393.1117	-2.1749	3.09E-21	5.98E-19	LOC110371345	glutathione S-transferase 1-like
1.1E+08	301.4734	560.2666	-0.89408	1.79E-07	5.96E-06	LOC110371688	cytochrome P450 6B5-like
1.1E+08	22499.59	17527.44	0.36028	0.0049906	0.040782	LOC110371725	cytochrome P450 6B5-like

1.1E+08	2034.849	1253.66	0.69878	9.10E-10	4.60E-08	LOC110371737	cytochrome P450 6B5-like
1.1E+08	23836.71	40567.35	-0.76713	3.82E-19	5.65E-17	LOC110371743	cytochrome P450 6B5-like%2C
1.1E+08	448.298	293.7164	0.61003	0.0009061	0.010558	LOC110371744	cytochrome P450 6B5-like
1.1E+08	1392.43	5085.748	-1.8689	7.42E-73	1.77E-69	LOC110371751	cytochrome P450 6B5-like
1.1E+08	5090.896	7410.797	-0.54171	7.71E-09	3.31E-07	LOC110372637	trypsin CFT-1-like
1.1E+08	2944.652	4821.694	-0.71144	1.04E-07	3.68E-06	LOC110372954	venom carboxylesterase-6-like%2C
1.1E+08	2.270779	42.00164	-4.2092	2.58E-05	0.0005147	LOC110373009	probable NADH dehydrogenase [ubiquinone] 1 alpha subcomplex subunit 12
1.1E+08	680.3136	1218.967	-0.84139	7.76E-10	4.00E-08	LOC110373289	glutathione S-transferase 1-like
1.1E+	2826.91	1335.272	1.0821	3.63E-2	8.97E-2	LOC11037	cytochrome P450 6B1-like

08				3	1	3339	
1.1E+<br>08	12.40875	97.68577	-2.9768	0.0048<br>601	0.0400<br>21	LOC11037<br>4025	chymotrypsin-like protease CTRL-1
1.1E+<br>08	19945.94	24625.06	-0.30403	0.0036<br>358	0.0322<br>98	LOC11037<br>4557	cytochrome P450 6B2-like
1.1E+<br>08	4929.27	7084.175	-0.52323	1.92E-0<br>8	7.61E-0<br>7	LOC11037<br>4762	cytochrome P450 CYP12A2-like
1.1E+<br>08	805.2486	7444.543	-3.2087	4.38E-2<br>03	6.27E-1<br>99	LOC11037<br>5046	trypsin%2C alkaline C-like
1.1E+<br>08	1659.854	2434.444	-0.55254	0.0010<br>521	0.0119<br>55	LOC11037<br>5101	glutathione S-transferase 1-1-like
1.1E+<br>08	27.93659	69.43844	-1.3136	0.0037<br>318	0.0329<br>67	LOC11037<br>5128	acetylcholine receptor subunit beta-like 1%2C
1.1E+<br>08	724.2914	461.6635	0.64973	0.0025<br>042	0.0239<br>38	LOC11037<br>5176	neuronal acetylcholine receptor subunit alpha-7-like%2C
1.1E+<br>08	755.1259	307.7443	1.295	1.02E-1<br>5	1.10E-1<br>3	LOC11037<br>5353	trypsin-1-like%2C

1.1E+08	330.3783	540.6892	-0.71068	3.81E-05	0.0007321	LOC110375658	chymotrypsin-2-like
1.1E+08	226.7057	343.4528	-0.59929	0.002231	0.021967	LOC110375713	cytochrome P450 18a1-like
1.1E+08	1471.662	3600.668	-1.2908	3.69E-34	1.82E-31	LOC110375842	acetylcholinesterase-like
1.1E+08	485.0094	931.904	-0.94217	9.92E-11	5.89E-09	LOC110375843	acetylcholinesterase-like
1.1E+08	302.1057	501.8596	-0.73223	2.86E-05	0.0005669	LOC110375844	acetylcholinesterase-like
1.1E+08	101294.6	134306.8	-0.40698	9.87E-07	2.83E-05	LOC110376134	trypsin CFT-1-like
1.1E+08	24.72751	74.93757	-1.5996	0.0005635	0.0072506	LOC110376142	trypsin%2C alkaline B-like
1.1E+08	13.74245	100.9155	-2.8764	6.59E-10	3.43E-08	LOC110376168	trypsin CFT-1-like
1.1E+	1058.364	469.4459	1.1728	2.54E-1	2.95E-1	LOC11037	probable cytochrome P450 49a1%2C

08				6	4	6697	
1.1E+08	2412.971	1034.868	1.2214	2.81E-24	7.58E-22	LOC110376698	probable cytochrome P450 301a1%2C mitochondrial
1.1E+08	1814.507	2245.895	-0.30771	0.0048422	0.03992	LOC110376856	trypsin%2C alkaline B-like%2C
1.1E+08	33.57475	4.488122	2.9032	0.0003833	0.0052389	LOC110377115	trypsin-7-like%2C
1.1E+08	5972.466	4764.853	0.32589	0.000551	0.0071089	LOC110377241	cytochrome P450 6B6-like
1.1E+08	3930.941	4877.916	-0.31139	0.0011608	0.012956	LOC110377393	cytochrome P450 4d2-like
1.1E+08	2225.619	836.7202	1.4114	2.16E-33	9.66E-31	LOC110377394	cytochrome P450 4d2-like
1.1E+08	7445.873	9455	-0.34464	0.0001517	0.0024061	LOC110377401	cytochrome P450 CYP12A2-like
1.1E+08	669.9854	496.8017	0.43146	0.005689	0.044889	LOC110377685	glutathione S-transferase C-terminal domain-containing protein homolog

1.1E+08	50.19211	252.6722	-2.3317	0.0023371	0.022613	LOC110377912	glutathione S-transferase 1-like%2C
1.1E+08	898.6089	532.9406	0.75372	1.63E-07	5.49E-06	LOC110378474	cytochrome P450 9e2-like%2C
1.1E+08	6146.375	3842.318	0.67776	0.0008552	0.010071	LOC110378476	cytochrome P450 9e2-like%2C
1.1E+08	1299.788	2116.435	-0.70336	2.77E-10	1.52E-08	LOC110378477	cytochrome P450 9e2-like%2C
1.1E+08	24.24195	149.4799	-2.6244	2.36E-07	7.64E-06	LOC110378558	trypsin%2C alkaline C-like
1.1E+08	2649.251	1835.188	0.52966	5.78E-07	1.72E-05	LOC110378861	cytochrome P450 4C1-like%2C
1.1E+08	2966.262	4514.056	-0.60578	7.82E-10	4.01E-08	LOC110378933	acetylcholinesterase-like
1.1E+08	1027.928	642.2275	0.67858	7.75E-07	2.26E-05	LOC110378938	cytochrome P450 4C1-like
1.1E+	1754.452	1419.89	0.30524	0.0065	0.0498	LOC11037	cytochrome P450 4C1-like

08				696	03	8939	
1.1E+08	11999.23	18858.37	-0.65226	1.32E-06	3.68E-05	LOC110378942	cytochrome P450 4C1-like
1.1E+08	7143.104	4141.326	0.78646	3.98E-06	9.92E-05	LOC110379024	trypsin%2C alkaline C-like
1.1E+08	1050.291	689.3503	0.60748	7.90E-06	0.0001831	LOC110379202	carboxylesterase 1E
1.1E+08	16808.29	22713.45	-0.43437	3.22E-07	1.02E-05	LOC110379569	chymotrypsin-1-like
1.1E+08	1336.56	2512.606	-0.91066	0.0001563	0.0024651	LOC110380060	trypsin-3-like
1.1E+08	937.5302	628.2866	0.57744	0.0028724	0.026571	LOC110380575	trypsin%2C alkaline C-like
1.1E+08	102.9056	515.5945	-2.3249	2.29E-20	3.96E-18	LOC110380579	trypsin%2C alkaline C-like
1.1E+08	556.6387	67.04078	3.0536	2.16E-11	1.46E-09	LOC110380583	trypsin CFT-1-like

1.1E+08	22154.78	81971.05	-1.8875	5.24E-82	2.50E-78	LOC110380587	trypsin%2C alkaline C-like
1.1E+08	91.85171	160.2366	-0.80283	0.0065098	0.049506	LOC110380833	cytochrome P450 4C1-like
1.1E+08	5.480083	59.69361	-3.4453	8.38E-08	2.99E-06	LOC110380839	cytochrome P450 4V2-like
1.1E+08	333.479	49.48613	2.7525	2.02E-25	6.01E-23	LOC110380841	cytochrome P450 4V2-like
1.1E+08	9657.198	2431.224	1.9899	1.63E-19	2.54E-17	LOC110381029	cytochrome P450 6B5-like
1.1E+08	958.3404	1576.872	-0.71846	1.44E-06	3.98E-05	LOC110381030	cytochrome P450 6B2-like
1.1E+08	636.889	406.8824	0.64643	4.80E-05	0.000895	LOC110381041	cytochrome P450 6B2-like
1.1E+08	433.5569	594.6296	-0.45577	0.0036504	0.032408	LOC110381376	cytochrome P450 4C1-like%2C
1.1E+	2220.986	1205.159	0.88198	1.66E-0	6.80E-0	LOC11038	probable cytochrome P450 6a13

08				8	7	2836	
1.1E+08	1.604652	18.73168	-3.5451	0.0031824	0.028898	LOC110383665	trypsin%2C alkaline C-like%2C
1.1E+08	1244.082	729.8068	0.7695	1.56E-09	7.59E-08	LOC110383729	glutathione S-transferase theta-1
1.1E+08	2543.829	1782.968	0.51272	1.55E-06	4.25E-05	LOC110383926	glutathione S-transferase 1-like
1.1E+08	2495.984	4123.39	-0.72422	1.13E-05	0.000247	LOC110383942	cytochrome P450 6k1-like
1.1E+08	5892.246	12396.94	-1.0731	2.37E-08	9.19E-07	LOC110383952	cytochrome P450 6l1-like
1.1E+08	9894.508	15622.82	-0.65896	1.64E-13	1.43E-11	LOC110383977	chymotrypsin-1-like%2C
1.1E+08	217.4467	410.4637	-0.91659	2.92E-06	7.51E-05	LOC110384045	trypsin-3-like
1.1E+08	355.6144	608.8407	-0.77575	2.66E-06	6.88E-05	LOC110384314	cytochrome P450 6B6-like

1.1E+08	44.6037	253.5003	-2.5068	5.14E-08	1.90E-06	LOC110384478	anionic trypsin-1-like
1.1E+08	58.54447	383.3552	-2.7111	0.000632	0.0079603	LOC110384517	probable cytochrome P450 301a1%2C mitochondrial
1.1E+08	505.0999	338.2305	0.57856	0.0009579	0.011071	LOC110384658	trypsin-1
1.1E+08	3.56698	50.68628	-3.8288	0.0005098	0.0066797	LOC110384679	trypsin epsilon-like

Table S2

Gene_id	readcount_C2-0.5 mg/mL	readcount_CK	log2FoldChange	pval	padj	Gene name	Description
1.1E+08	1389.691	1034.439	0.42592	0.0017979	0.0065641	LOC110369693	glutathione S-transferase 1-like%2C
1.1E+08	9441.937	6567.351	0.52377	4.47E-14	5.25E-13	LOC110369738	glutathione S-transferase 1-like%2C
1.1E+08	9251.592	7995.973	0.21043	0.0021887	0.0078175	LOC110369962	NADH dehydrogenase [ubiquinone] 1 beta subcomplex subunit 11%2C mitochondrial
1.1E+08	47277.87	30356.78	0.63915	2.97E-24	6.32E-23	LOC110370005	NADPH--cytochrome P450 reductase%2C
1.1E+08	2214.965	1473.122	0.58841	1.62E-05	8.37E-05	LOC110370104	cytochrome P450 6B6-like
1.1E+08	8347.284	13626.43	-0.70703	1.05E-23	2.18E-22	LOC110370167	gamma-aminobutyric acid receptor-associated protein
1.1E+	2492.332	3297.26	-0.40377	1.02E	6.23E	LOC110	cytochrome P450 6B6-like

08				-06	-06	370521	
1.1E+08	69.8695	344.6478	-2.3024	1.12E-22	2.19E-21	LOC110371001	acetylcholinesterase-like
1.1E+08	5981.795	5096.17	0.23116	0.001747	0.006393	LOC110371115	NADH dehydrogenase [ubiquinone] flavoprotein 1%2C mitochondrial-like
1.1E+08	8365.274	7250.204	0.20639	0.0027004	0.00947	LOC110371229	NADH dehydrogenase [ubiquinone] iron-sulfur protein 4%2C mitochondrial
1.1E+08	792.4898	375.9805	1.0757	9.18E-15	1.13E-13	LOC110371334	glutathione S-transferase 1-like
1.1E+08	48.12156	15.28446	1.6546	0.0031205	0.010826	LOC110371341	glutathione S-transferase 1-like
1.1E+08	28618.34	23148.61	0.30601	2.11E-06	1.24E-05	LOC110371343	glutathione S-transferase 1-like%2C
1.1E+08	1670.45	903.4036	0.88679	4.21E-17	6.19E-16	LOC110371344	glutathione S-transferase 1-like
1.1E+08	144.723	404.2053	-1.4818	7.77E-14	8.97E-13	LOC110371345	glutathione S-transferase 1-like

1.1E+ 08	21440.15	14942.5 1	0.52089	5.29E -16	7.20E -15	LOC110 371687	cytochrome P450 6B5-like
1.1E+ 08	40717.7	18029.6 5	1.1753	1.47E -74	1.10E -72	LOC110 371725	cytochrome P450 6B5-like
1.1E+ 08	1093.732	511.450 9	1.0966	4.90E -08	3.50E -07	LOC110 371731	cytochrome P450 6B5-like
1.1E+ 08	346.9257	144.058 1	1.268	6.32E -10	5.40E -09	LOC110 371736	cytochrome P450 6B5-like
1.1E+ 08	2658.045	1289.02 5	1.0441	2.77E -14	3.31E -13	LOC110 371737	cytochrome P450 6B5-like
1.1E+ 08	537.4	301.701 6	0.83287	2.67E -07	1.75E -06	LOC110 371744	cytochrome P450 6B5-like
1.1E+ 08	3167.393	5230.94 7	-0.72378	1.03E -05	5.48E -05	LOC110 371751	cytochrome P450 6B5-like
1.1E+ 08	7853.309	3911.62 4	1.0055	9.82E -16	1.31E -14	LOC110 371777	cytochrome P450 4g15-like
1.1E+	5374.742	2709.85	0.98798	5.45E	1.79E	LOC110	cytochrome P450 4g15-like

08		8		-36	-34	371778	
1.1E+08	329.4292	798.9625	-1.2782	1.01E-18	1.64E-17	LOC110372238	probable cytochrome P450 49a1
1.1E+08	19985.53	16552.58	0.2719	2.24E-05	0.0001131	LOC110372386	NADH dehydrogenase [ubiquinone] 1 alpha subcomplex subunit 9%2C mitochondrial
1.1E+08	10322.28	7625.526	0.43685	6.83E-06	3.73E-05	LOC110372637	trypsin CFT-1-like
1.1E+08	143.7006	41.9799	1.7753	5.13E-08	3.66E-07	LOC110373169	glutathione S-transferase 2-like
1.1E+08	586.144	204.3703	1.5201	1.34E-15	1.78E-14	LOC110373170	glutathione S-transferase 2-like
1.1E+08	81480.58	55587.23	0.5517	5.85E-19	9.58E-18	LOC110373172	glutathione S-transferase 2-like
1.1E+08	1705.588	413.5229	2.0442	1.23E-68	8.64E-67	LOC110373211	glutathione S-transferase 2-like
1.1E+08	222.8205	1254.836	-2.4935	3.25E-34	1.01E-32	LOC110373289	glutathione S-transferase 1-like

1.1E+08	948.9644	586.8401	0.69339	2.15E-08	1.60E-07	LOC110373300	glutathione S-transferase S1-like
1.1E+08	10.38279	1373.584	-7.0476	1.36E-149	2.56E-147	LOC110373339	cytochrome P450 6B1-like
1.1E+08	33.32507	195.6858	-2.5539	3.53E-16	4.87E-15	LOC110373494	venom carboxylesterase-6
1.1E+08	5076.663	3535.908	0.5218	8.42E-12	8.32E-11	LOC110373767	NADH dehydrogenase [ubiquinone] 1 alpha subcomplex subunit 8
1.1E+08	130756.7	79912.2	0.7104	2.00E-30	5.45E-29	LOC110374239	catalase
1.1E+08	2003.504	1701.721	0.23553	0.0097218	0.029919	LOC110374274	venom carboxylesterase-6-like
1.1E+08	8563.167	7296.921	0.23086	0.0006004	0.0024059	LOC110374470	NADH dehydrogenase [ubiquinone] 1 beta subcomplex subunit 8%2C mitochondrial
1.1E+08	37777.68	25326.66	0.57688	1.84E-10	1.64E-09	LOC110374557	cytochrome P450 6B2-like
1.1E+	1038.369	509.547	1.027	3.11E	1.78E	LOC110	catalase-like

08				-06	-05	374563	
1.1E+<br>08	8569.385	7285.71<br>3	0.23412	0.009<br>1564	0.028<br>417	LOC110<br>374762	cytochrome P450 CYP12A2-like
1.1E+<br>08	2204.628	7657.40<br>5	-1.7963	2.19E<br>-118	3.01E<br>-116	LOC110<br>375046	trypsin%2C alkaline C-like
1.1E+<br>08	63849.65	40917.8<br>9	0.64195	1.46E<br>-24	3.14E<br>-23	LOC110<br>375100	glutathione S-transferase 1-like
1.1E+<br>08	67828.57	28652.3<br>7	1.2432	1.64E<br>-83	1.44E<br>-81	LOC110<br>375102	glutathione S-transferase 1-like
1.1E+<br>08	10.27754	71.2034<br>6	-2.7925	3.48E<br>-07	2.24E<br>-06	LOC110<br>375128	acetylcholine receptor subunit beta-like 1%2C
1.1E+<br>08	70.39424	474.646<br>5	-2.7533	9.62E<br>-24	2.01E<br>-22	LOC110<br>375176	neuronal acetylcholine receptor subunit alpha-7-like%2C
1.1E+<br>08	4551.308	3881.72<br>7	0.22958	0.003<br>0027	0.010<br>463	LOC110<br>375180	microsomal glutathione S-transferase 1-like
1.1E+<br>08	0.946073	316.516<br>4	-8.3861	3.10E<br>-74	2.31E<br>-72	LOC110<br>375353	trypsin-1-like%2C

1.1E+<br>08	4934.387	3271.86<br>1	0.59276	2.97E<br>-13	3.28E<br>-12	LOC110<br>375476	probable NADH dehydrogenase [ubiquinone] iron-sulfur protein 6%2C mitochondrial
1.1E+<br>08	6075.572	4937.29	0.2993	0.000<br>1964	0.000<br>8577	LOC110<br>375532	NADH dehydrogenase [ubiquinone] flavoprotein 2%2C mitochondrial
1.1E+<br>08	370.2131	741.123<br>5	-1.0014	2.83E<br>-12	2.89E<br>-11	LOC110<br>375691	cytochrome P450 18a1-like
1.1E+<br>08	93.94274	352.818<br>2	-1.9091	2.48E<br>-18	3.96E<br>-17	LOC110<br>375713	cytochrome P450 18a1-like
1.1E+<br>08	12223.05	9978.95<br>6	0.29264	1.17E<br>-05	6.19E<br>-05	LOC110<br>375798	NADH dehydrogenase [ubiquinone] 1 beta subcomplex subunit 5%2C mitochondrial
1.1E+<br>08	354.0648	515.897<br>3	-0.54307	0.000<br>5533	0.002<br>2341	LOC110<br>375844	acetylcholinesterase-like
1.1E+<br>08	767.6632	584.194<br>2	0.39403	0.002<br>4065	0.008<br>527	LOC110<br>375888	cytochrome P450 9e2-like
1.1E+<br>08	5773.834	4845.52<br>6	0.25288	0.000<br>428	0.001<br>7584	LOC110<br>375902	NADH dehydrogenase [ubiquinone] 1 alpha subcomplex subunit 6-like
1.1E+	245.5543	77.0579	1.672	3.30E	0.000	LOC110	trypsin%2C alkaline B-like

08		4		-05	1622	376142	
1.1E+ 08	432.9599	103.807 7	2.0603	7.98E -13	8.59E -12	LOC110 376168	trypsin CFT-1-like
1.1E+ 08	13220.08	10752.8 9	0.29801	5.47E -06	3.03E -05	LOC110 376300	NADH dehydrogenase [ubiquinone] iron-sulfur protein 3%2C mitochondrial
1.1E+ 08	6.084425	35.1755 3	-2.5314	0.000 5991	0.002 4025	LOC110 376517	cytochrome P450 9e2-like
1.1E+ 08	4.773651	51.8946 8	-3.4424	2.01E -07	1.33E -06	LOC110 376530	cytochrome P450 9e2-like
1.1E+ 08	3182.058	2691.73 1	0.24143	0.003 2174	0.011 119	LOC110 376581	NADH dehydrogenase [ubiquinone] 1 alpha subcomplex subunit 2
1.1E+ 08	334.6649	482.672 7	-0.52833	0.000 8502	0.003 3159	LOC110 376697	probable cytochrome P450 49a1%2C
1.1E+ 08	207.426	1063.79 4	-2.3586	1.28E -59	7.58E -58	LOC110 376698	probable cytochrome P450 301a1%2C mitochondrial
1.1E+ 08	7945.469	12676.8 8	-0.674	3.56E -23	7.19E -22	LOC110 376988	lysosome-associated membrane glycoprotein 2-like

1.1E+08	715.027	556.7888	0.36087	0.0075746	0.024045	LOC110377156	glutathione S-transferase 1-like
1.1E+08	4655.342	2851.757	0.70704	7.88E-19	1.28E-17	LOC110377184	glutathione S-transferase 1-1
1.1E+08	1728.837	4896.809	-1.502	2.98E-23	6.06E-22	LOC110377241	cytochrome P450 6B6-like
1.1E+08	6772.505	5016.345	0.43305	1.54E-07	1.04E-06	LOC110377393	cytochrome P450 4d2-like
1.1E+08	534.4259	861.3208	-0.68856	3.44E-06	1.96E-05	LOC110377394	cytochrome P450 4d2-like
1.1E+08	4934.521	9724.467	-0.97871	4.20E-42	1.62E-40	LOC110377401	cytochrome P450 CYP12A2-like
1.1E+08	11791.52	9873.391	0.25613	9.69E-05	0.0004437	LOC110377463	NADH dehydrogenase [ubiquinone] 1 beta subcomplex subunit 10
1.1E+08	6259.861	4306.119	0.53974	1.80E-13	2.01E-12	LOC110377624	NADH dehydrogenase [ubiquinone] 1 beta subcomplex subunit 9
1.1E+	936.3154	510.784	0.87428	1.72E	1.65E	LOC110	glutathione S-transferase C-terminal domain-containing protein

08		3		-11	-10	377685	homolog
1.1E+08	401.2361	547.7855	-0.44916	0.0030575	0.010631	LOC110378474	cytochrome P450 9e2-like%2C
1.1E+08	1846.657	2175.677	-0.23655	0.011525	0.034956	LOC110378477	cytochrome P450 9e2-like%2C
1.1E+08	39.64877	153.7865	-1.9556	2.01E-05	0.0001024	LOC110378558	trypsin%2C alkaline C-like
1.1E+08	7744.149	4645.199	0.73737	9.22E-25	2.00E-23	LOC110378933	acetylcholinesterase-like
1.1E+08	2107.129	1460.254	0.52906	2.08E-07	1.38E-06	LOC110378939	cytochrome P450 4C1-like
1.1E+08	17108.81	4250.191	2.0091	6.13E-18	9.49E-17	LOC110379024	trypsin%2C alkaline C-like
1.1E+08	79.81336	708.8344	-3.1507	5.63E-61	3.43E-59	LOC110379202	carboxylesterase 1E
1.1E+08	11740.68	23351.73	-0.99201	1.85E-10	1.65E-09	LOC110379569	chymotrypsin-1-like

1.1E+08	73778.97	42133.22	0.80825	2.66E-37	8.97E-36	LOC110379731	antichymotrypsin-1-like%2C
1.1E+08	9.278928	372.6611	-5.3278	3.63E-66	2.45E-64	LOC110379849	trypsin-1-like%2C
1.1E+08	4958.012	4370.648	0.18191	0.012549	0.037678	LOC110379902	probable NADH dehydrogenase [ubiquinone] 1 alpha subcomplex subunit 12
1.1E+08	7712.403	10002.14	-0.37506	7.44E-08	5.20E-07	LOC110380413	cytochrome P450 6B6-like
1.1E+08	155.1756	530.7145	-1.774	6.52E-14	7.57E-13	LOC110380579	trypsin%2C alkaline C-like
1.1E+08	44.05605	148.4801	-1.7529	9.36E-08	6.48E-07	LOC110380581	trypsin CFT-1-like
1.1E+08	1106.418	2391.7	-1.1121	2.06E-11	1.96E-10	LOC110380582	trypsin%2C alkaline C-like
1.1E+08	125.2679	68.89362	0.86257	0.0076739	0.024312	LOC110380583	trypsin CFT-1-like
1.1E+	8.295159	94.4267	-3.5089	2.96E	3.01E	LOC110	trypsin%2C alkaline C-like

08		5		-12	-11	380584	
1.1E+08	102219.9	118021.5	-0.20737	0.015446	0.045209	LOC110380585	trypsin CFT-1-like
1.1E+08	30108.97	84269.69	-1.4848	2.90E-89	2.79E-87	LOC110380587	trypsin%2C alkaline C-like
1.1E+08	1588.152	2350.631	-0.5657	4.24E-10	3.68E-09	LOC110380735	chymotrypsin-like elastase family member 2A%2C
1.1E+08	375.1084	164.8359	1.1863	1.41E-09	1.17E-08	LOC110380833	cytochrome P450 4C1-like
1.1E+08	128.9931	52.78226	1.2892	0.0093317	0.02888	LOC110380838	cytochrome P450 4C1-like
1.1E+08	145.3418	61.30019	1.2455	0.0002068	0.0008988	LOC110380839	cytochrome P450 4V2-like
1.1E+08	11.47464	50.88905	-2.1489	0.000194	0.0008493	LOC110380841	cytochrome P450 4V2-like
1.1E+08	5162.251	9120.05	-0.82104	2.18E-25	4.92E-24	LOC110380950	acetylcholinesterase 1-like

1.1E+08	1913.201	2501.087	-0.38657	1.70E-05	8.79E-05	LOC110381029	cytochrome P450 6B5-like
1.1E+08	1209.023	1622.859	-0.4247	4.69E-05	0.0002247	LOC110381030	cytochrome P450 6B2-like
1.1E+08	3797.066	1701.51	1.1581	5.56E-36	1.82E-34	LOC110381048	cytochrome P450 6k1-like
1.1E+08	1282.29	611.466	1.0684	1.16E-19	1.96E-18	LOC110381376	cytochrome P450 4C1-like%2C
1.1E+08	470.3911	168.3475	1.4824	2.22E-13	2.47E-12	LOC110381377	cytochrome P450 4C1-like
1.1E+08	2947.259	1610.511	0.87186	1.08E-22	2.12E-21	LOC110381380	cytochrome P450 4V2-like
1.1E+08	16180.7	12126.06	0.41616	9.64E-10	8.10E-09	LOC110381963	NADH dehydrogenase [ubiquinone] 1 alpha subcomplex subunit 10%2C mitochondrial
1.1E+08	12042	1240.004	3.2797	0	0	LOC110382836	probable cytochrome P450 6a13
1.1E+	8626.886	6746.47	0.35471	0.000	0.000	LOC110	NADH dehydrogenase [ubiquinone] 1 beta subcomplex subunit 3

08		9		1275	5747	382887	
1.1E+<br>08	6338.899	3230.08<br>2	0.97266	2.84E<br>-08	2.09E<br>-07	LOC110<br>383666	trypsin%2C alkaline C-like
1.1E+<br>08	3066.765	2275.25<br>5	0.43069	0.000<br>1001	0.000<br>4579	LOC110<br>383804	biogenesis of lysosome-related organelles complex 1 subunit 5-like%2C
1.1E+<br>08	2550.39	1908.10<br>4	0.41858	1.24E<br>-06	7.55E<br>-06	LOC110<br>383833	venom carboxylesterase-6-like
1.1E+<br>08	1235.902	1832.66<br>5	-0.56838	6.25E<br>-09	4.89E<br>-08	LOC110<br>383926	glutathione S-transferase 1-like
1.1E+<br>08	20722.12	12757.2<br>6	0.69985	4.06E<br>-10	3.52E<br>-09	LOC110<br>383952	cytochrome P450 6l1-like
1.1E+<br>08	6737.088	5484.81<br>4	0.29668	2.97E<br>-05	0.000<br>1468	LOC110<br>383955	NADH dehydrogenase [ubiquinone] 1 beta subcomplex subunit 2%2C mitochondrial-like
1.1E+<br>08	9864.506	16072.4<br>8	-0.70427	8.47E<br>-13	9.09E<br>-12	LOC110<br>383977	chymotrypsin-1-like%2C
1.1E+<br>08	1571.75	2390.24<br>6	-0.60479	3.56E<br>-11	3.34E<br>-10	LOC110<br>384037	trypsin alpha-4-like%2C

1.1E+08	6810.81	5544.773	0.2967	2.48E-05	0.0001245	LOC110384117	NADH dehydrogenase [ubiquinone] 1 beta subcomplex subunit 7
1.1E+08	397.5737	195.8521	1.0215	5.59E-08	3.96E-07	LOC110384152	glutathione S-transferase 1-like%2C
1.1E+08	1023.547	119.1872	3.1023	1.60E-46	7.04E-45	LOC110384153	glutathione S-transferase 1-like
1.1E+08	1151.743	109.15	3.3994	1.97E-92	1.97E-90	LOC110384165	cytochrome P450 4C1-like
1.1E+08	8531.126	4566.836	0.90154	4.56E-36	1.50E-34	LOC110384246	cytochrome P450 6B2
1.1E+08	8407.057	3349.084	1.3278	5.64E-37	1.89E-35	LOC110384247	cytochrome P450 6B7
1.1E+08	2490.233	1745.038	0.51302	0.0016478	0.0060688	LOC110384248	cytochrome P450 6B6
1.1E+08	1670.736	261.2781	2.6768	8.55E-12	8.43E-11	LOC110384478	anionic trypsin-1-like
1.1E+	12.12974	347.471	-4.8403	8.87E	5.10E	LOC110	trypsin-1

08		8		-58	-56	384658	
Novel 00242	40347.27	29224.43	0.4653	0.0004395	0.0018019	--	trypsin-like protease [Helicoverpa armigera]>gi|297340778|gb|ADI32887.1| serine protease [Helicoverpa armigera]
